# Supplementary material for: Diabetes Control Status and Severity of Depression: Insights from NHANES 2005–2020
Source: Biomedicines. 2024 Oct 8;12(10):2276. doi: 10.3390/biomedicines12102276 (PMC11504683; doi:10.3390/biomedicines12102276)
Supplement: Supplementary file 1 [file biomedicines-12-02276-s001.zip › biomedicines-3143880-supplementary.pdf]

Appendix SA:

The CDC website for the NHANES data description used a Physical Activity Questionnaire (variable name prefix PAQ), based on the Global Physical Activity Questionnaire (GPAQ) to assess the participant level of physical activities.

The calculation of MET Score was done using the following data which were derived from the NHANES website. *https://wwwn.cdc.gov/nchs/nhanes/search/datapage.aspx?Component=Questionnaireand the codebook and analytical notes were referred to for further details.

Column Corresponding to Yes/No for the Activity	Column Corresponding to Typical Number of Days per Week Allotted for the Activity	Column Corresponding to Typical Duration in Minutes per Day for the Activity	Label	Score
PAQ605	PAQ610	PAD615	Vigorous work-related activity	8.0
PAQ620	PAQ625	PAD630	Moderate work-related activity	4.0
PAQ635	PAQ640	PAD645	Walking or bicycling for transportation	4.0
PAQ650	PAQ655	PAD660	Vigorous leisure-time physical activity	8.0
PAQ665	PAQ670	PAD675	Moderate leisure-time physical activity	4.0

Using this information, the datasets were cleaned, and new columns were created to (1) calculate the Metabolic Equivalent of Task (MET) Minutes per Week for different activities, (2) summate the MET Score, and (3) compute the total MET Hours per Week for these activities.

Calculation of MET Score – Years 2007-2020:

If the column corresponding to the Yes/No question for the activity had a value of 2 (No), they were placed in the lowest category (MET-hours/week = 0). For the answers of 7 (Refused), 9 (Don’t Know), or missing, the data related to the column was considered missing. If the column had a value of 1 (Yes), then the following calculation was done:

*MET Minutes per Week for Each Activity = Column Corresponding to Typical Number of Days per Week Allotted for the Activity * Column Corresponding to Typical Duration in Minutes per Day for the Activity * Given MET Score for Each Activity*

If any of the columns corresponding to the typical number of days per week allotted for the activity had a value of 77 (Refused), 99 (Don't Know), or missing, then again, the data related to the column was considered missing. Similarly, if the columns corresponding to the typical duration in minutes per day for the activity had a value of 7777 (Refused), 9999 (Don't Know), or missing, the data related to the column was considered missing. Otherwise, the real value was considered for calculation.

Sum MET Score = ∑ MET Minutes for Each Activity Excluding Missing Values

MET Hours per Week = Sum MET Score / 60

Finally, after the MET Hours per Week was obtained, the data was divided into groups. Participants with no physical activity (MET-hours/week = 0) were placed in the "No Activity" category, while the others were divided into four categories (Quartiles 1-4), and a separate category was created for participants with missing data.

Calculation of MET Score – Years 2005-2006:

Since Physical Activity was a covariate of the analysis, the correlation of the 2005-2006 dataset with the rest of the years, was done using the question PAQ180 - Average level of physical activity each day, as follows:

Physical Activity Questionnaire	Description	MET Score
PAQ180	Average level of physical activity each day (1 - Mainly sit)	1.4
PAQ180	Average level of physical activity each day (2 - Walk a lot)	1.5
PAQ180	Average level of physical activity each day (3 - Carry light loads)	1.6
PAQ180	Average level of physical activity each day (4 - Carry heavy loads)	1.8

This table was retrieved from Appendix – 1: Suggested MET Scores for the years 2005-2006.
*https://wwwn.cdc.gov/Nchs/Nhanes/2005-2006/PAQ_D.htm#Appendix_1._Suggested_MET_Scores

If any of the columns corresponding to the average level of physical activity had a value of 7 (Refused), 9 (Don't Know), or missing, then the data related to the column was considered missing. Otherwise, using the above-mentioned MET Scores, the participants with MET Score = 1.4 corresponded to the "Qartile 1" , MET Score = 1.5 to the "Qartile 2", MET Score = 1.6 to the "Qartile 3", and MET Score = 1.8 to the "Qartile 4", and a separate category was created for participants with missing data.
